# Dogs’ Eavesdropping from People’s Reactions in Third Party Interactions

**DOI:** 10.1371/journal.pone.0079198

**Published:** 2013-11-13

**Authors:** Esteban Freidin, Natalia Putrino, María D’Orazio, Mariana Bentosela

**Affiliations:** 1 Centro de Recursos Naturales Renovables de la Zona Semiárida, Centro Científico Tecnológico Consejo Nacional de Investigaciones Científicas y Técnicas, Bahía Blanca, Argentina; 2 Grupo de Investigación del Comportamiento en Cánidos, Instituto de Investigaciones Médicas Alfredo Lanari, Consejo Nacional de Investigaciones Científicas y Técnicas/Universidad de Buenos Aires, Buenos Aires, Argentina; Harvard University, United States of America

## Abstract

Eavesdropping involves the acquisition of information from third-party interactions, and can serve to indirectly attribute reputation to individuals. There is evidence on eavesdropping in dogs, indicating that they can develop a preference for people based on their cooperativeness towards others. In this study, we tested dogs’ eavesdropping abilities one step further. In a first experiment, dogs could choose between cooperative demonstrators (the donors) who always gave food to an approaching third person (the beggar); here, the only difference between donors was whether they received positive or negative reactions from the beggar (through verbal and gestural means). Results showed that dogs preferentially approached the donor who had received positive reactions from the beggar. By contrast, two different conditions showed that neither the beggar’s body gestures nor the verbal component of the interaction on their own were sufficient to affect the dogs’ preferences. We also ran two further experiments to test for the possibility of dogs’ choices being driven by local enhancement. When the donors switched places before the choice, dogs chose at random. Similarly, in a nonsocial condition in which donors were replaced by platforms, subjects chose at chance levels. We conclude that dogs’ nonrandom choices in the present protocol relied on the simultaneous presence of multiple cues, such as the place where donors stood and several features of the beggar’s behavior (gestural and verbal reactions, and eating behavior). Nonetheless, we did not find conclusive evidence that dogs discriminated the donors by their physical features, which is a prerequisite of reputation attribution.

## Introduction

Social learning allows animals to acquire valuable information by observing or interacting with other organisms without having to incur the costs of individual trial-and-error [1]–[3]. One particular instance of social learning is called eavesdropping which involves the extraction of information from the interaction between third parties [4]. Eavesdropping can have important evolutionary consequences not only because of its direct fitness consequences to the eavesdropper, but also because it may change the payoffs involved in interactions with and without an audience. This may be relevant in diverse domains, from sexual selection and animal contests to reciprocity and cooperation [5]. Researchers describe a variety of behaviors that can be considered cases of eavesdropping in fish, birds, and mammals. For example, Bshary and Grutter [6] found eavesdropping in a cleaning mutualism involving the cleaner fish *Labroides dimidiatus*. In this system, bystander clients find cooperative partners and thus gain personal benefits from observing the interactions of other clients with more or less cooperative cleaners (those who eat the client’s ectoparasites as opposed to its mucus) [6]. Other examples involve the use of sexual signals to choose high quality foster parents’ by parasitic birds [4], and chimpanzees’ preference for persons who give food to a third individual relative to persons who do not [7].

Here we are concerned with the possibility of eavesdropping in dogs while they observe interactions among people. Domestic dogs (*Canis familiaris*) are a particularly suitable species to study eavesdropping because of at least two reasons. First, dogs have been through a domestication process that is estimated to have lasted at least for 15,000 years (e.g. [8]; but probably for much longer, [9]). Domestication is presumed to have led dogs to evolve adaptations to human environments. More specifically, some authors claim that the socio-cognitive abilities of dogs, in particularly those involved in “reading” human communicative cues, may be a consequence of the domestication process [10], [11]. An instance of these abilities is represented by dogs’ discrimination of human emotions. For example, dogs have been shown to discriminate a human approaching in a friendly as opposed to a threatening manner [12], and to prefer a person who gave them social rewards such as petting and positive verbalizations relative to an indifferent person [13]. Even more, dogs can discriminate between a smiling face and a neutral face [14], between an expression of happiness and one of disgust [15], and they can use that information to find food. Last, dogs recognize sad reactions in people and approach a crying person relative to another who is speaking or singing [16]. Nevertheless, authors do not agree about the origin of these abilities, and whether dogs’ communicative capacities are innate, learned, or result from an interaction of innate predispositions and learning [17], [18]. Second, domestic dogs live in intimate contact with people throughout their lives, thus having innumerable opportunities to learn about their behavior through direct and indirect interactions with them. In fact, dogs have been shown to be proficient at performing observational learning from human models [19], [20]. Moreover, people are dogs’ main providers of valuable resources, such as food, water, and shelter, and, given that people may vary in their disposition to cooperate and help others [21], dogs may benefit from discriminating between more or less cooperative types. Therefore, given dogs’ phylogenetic domestication history and their ontogeny in human contexts, we expect them to be strong candidates for sophisticated eavesdropping from humans.

Recent evidence suggests that domestic dogs may be capable of developing a preference for or against people they observe interacting with a third party. Rooney and Bradshaw [22] observed that dogs preferred to approach the winner as opposed to the loser of a tug-of-war game between a person and a demonstrator dog, suggesting that winners of games are perceived as desirable social partners. In turn, Kundey and collaborators [23] explored eavesdropping in a protocol in which dogs watched a “generous” demonstrator and a “selfish” demonstrator (both humans) interacting with a person asking for food (the beggar). The “generous” demonstrator consistently gave food to the beggar, whereas the “selfish” demonstrator consistently withheld food from the beggar. When released, all dogs showed a preference for the generous demonstrator over the selfish demonstrator. This finding was robust even when demonstrators switched places before the dog’s choice, thus controlling for local enhancement (experiment 6; [23]), and suggesting that dogs can develop a preference for people based on observation and indirect experience. To assess the interaction component, Marshall-Pescini and collaborators [24] did a similar experiment, but incorporating a phantom control group. In this control group, demonstrators performed the same behaviors as those done in the study by Kundey et al. [23], but without the presence of the beggar (i.e., in a noninteractive context). Indeed, dogs in the phantom control group showed no preference between demonstrators suggesting that in the interactive condition they formed their preference based on information obtained by observing the interaction and not on the demonstrators’ behavior alone [24]. Unfortunately, food was given only by one of the demonstrators and demonstrators never switched places in the study by Marshall-Pescini et al.; hence, local enhancement cannot be discarded as an alternative explanation to eavesdropping.

Last, the study by Nitzschner et al. [13] points towards some limitations on the conditions in which dogs may show eavesdropping. On one hand, these authors showed that dogs preferred to approach a “nice” person (who played with, talked to and stroked the subject) relative to an “ignoring” person based on previous direct interactions with them. On the other hand, these authors did not observe any preference after the subjects watched interactions between “nice” and “ignoring” demonstrators with another dog (experiment 2). Interestingly, subjects looked longer towards the “nice” than the “ignoring” experimenter during demonstrations in Nitzschner et al.’s experiment 2. However, the experimenter and the demonstrator dog spent more time together in the “nice” demonstration than in the “ignoring” demonstration. It is possible that the presence of the demonstrator dog could have overshadowed the attention towards the experimenter, especially in “nice” sessions, thus preventing the development of a strong preference. Moreover, in contrast with the data obtained by Kundey et al. [23] and by Marshall-Pescini et al. [24], results from Nitzschner et al. [13] suggest that the use of social reinforcement, instead of food, might make it harder for dogs to form a preference for people they observe interacting with third parties. Indeed, similar conclusions have been reached from dogs’ performance in other tasks [25], [26].

In sum, the evidence for eavesdropping in dogs is suggestive but not conclusive. The goal of the present study is to search for complementary data to build on a stronger case for dogs’ eavesdropping and their sophisticated socio-cognitive abilities. In the studies by Rooney and Bradshaw [22], Kundey et al. [23], Marshall-Pescini et al. [24], and Nitzschner et al. [13], authors focused on the information dogs could obtain from observing the demonstrators’ behavior during an interaction with a third party. However, they did not systematically vary the third party’s reaction to the demonstrators. We believe that third party’s reactions during interactions could serve as relevant cues of the payoffs implicated in the exchange (positive, neutral, or negative, and their magnitude) and of the cooperative quality of those involved. In experiment 1a, we assessed whether dogs could develop a preference between demonstrators (the donors) who behaved similarly but, towards which, a person asking for food (the beggar) reacted either positively or negatively through gestural (hand and body movements) and verbal means. The development of such a preference would imply that dogs should be capable of, first, discriminating the beggar’s positive and negative emotional reactions, second, associating those reactions with the corresponding donor, and third, using that information to choose which donor to beg food from. In addition, we tested two other conditions in which subjects only experienced either the gestural or the verbal component of the beggar’s reaction to assess dogs’ sensitivity to the different cues present in the main treatment. We also ran two follow-up experiments (1b and 1c) to control for and assess, respectively, a potential conditioning to the place, instead of to the donors. In the local enhancement control group (experiment 1b), the donors switched places in between demonstrations and before the dog could choose. In the phantom control group (experiment 1c), the beggar presented the same verbal and gestural cues of the main treatment from experiment 1a, though in a situation without donors (i.e., without the social interactive component).

## Experiment 1a

### Methods

In Argentina there is no special approval required for the use of dogs in social behavior and cognition studies in which there are no invasive or stressful manipulations. In any case, we consulted the Institutional Committee for Care and Use of Experimental Animals (CICUAL) of the Veterinary Sciences School, University of Buenos Aires. This study was carried out in strict accordance with the ethical standards of the CICUAL and complied with the current law of animal protection of Argentina (Law 14346). We obtained expressed consent from all owners for the participation of their dogs in this study.

**Subjects.** We recruited domestic dogs (*Canis familiaris*) by contacting and coordinating with their owners. We tested 72 dogs, with a mean (±1 SD) age of 4.73 (±2.83) years; 41 were male and 31 were female. In terms of breed, there were 17 Poodles, 5 German Shepherds, 5 Labrador Retrievers, 3 Golden Retrievers, 2 Cockers, 1 Beagle, 1 Border Collie, 1 Boxer, 1 Breton, 1 Dalmatian, 1 Fox Terrier, 1 French Bulldog, 1 Great Dane, 1 Pitbull Terrier, 1 Samoyed, 1 Shitzu, 1 Weimaraner, 1 Yorkshire, and 27 dogs of mixed breeds. Thirty six subjects had previous experience in other experimental communicative tasks.

Subjects were randomly assigned to one of three possible groups: group with gestural and verbal cues (GV, n = 23), group with gestural cues only (G, n = 26), and group with verbal cues only (V, n = 23).

**Figure 1:**
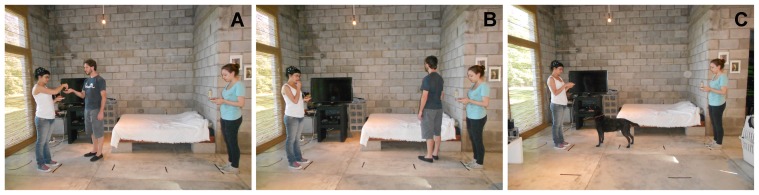
Photos of the experimental set and procedure. (A) The beggar receives food from the positive donor. (B) The beggar turns his back to the negative donor after having rejected the food. (C) The dog chooses the positive donor after the beggar left the room. All persons that appear in this figure have given written informed consent, as outlined in the PLOS consent form, to publication of their photograph.

**Materials.** We tested subjects individually in a familiar environment, be it their home or a dog care facility where they periodically attended. Once we were at the location, first, we prepared the experimental setup (we put the camera in the right position and marked the floor with tape) which took 5–10 min. During this time, the dog could only interact with the assistant that would later act as the dog handler (the other three assistants involved in the experimental situation did not interact with the dog). Second, the two assistants who acted as “donors”, took their positions, standing facing each other at a distance of 2 m (see Figure 1; the fourth assistant acted as the “beggar”). Donors were always female, and the beggar was the same male in all sessions. Each donor had a plate with pieces of sausage and corn flakes (sausages had a strong smell and were used to call dogs’ attention to the scene, whereas corn flakes were eaten by the donors and used to feed the beggar during demonstrations). A squared-shape “choice area” of 75 cm per side was marked in the floor around each donor. The dog was held 2 m from the intermediate point between the two donors, thus forming a triangle. The camera was attached to a tripod and located behind the dog in order to capture the choice area around each donor. The owner was not present in the room during the experiment.

**Procedure.** In the beginning of a session, the dog was held in the starting position, and the two donors approached him/her and showed their plates with sausages and corn flakes. The dog could smell the food for a few seconds but was not allowed to eat it. Then, the donors walked back to their respective positions (see Figure 1) and started eating the corn flakes at a regular pace (one flake every 5 sec), always directing their gaze towards the plate, and ignoring the dog at all times. The beggar was standing a meter behind the intermediate point between the donors’ positions (i.e., opposite of the dog’s starting position) and became active 10 seconds after the donors took their corresponding places. Once active, the beggar approached each donor three times (i.e., six interactions in total) in a random sequence with the provision that the he did not approach the same donor more than twice in a row. After approaching a donor, the beggar returned to his starting position from which he made his next approach. Once he was done with the 6^th^ interaction, he left the room.

In group GV, when approaching a donor, the beggar extended his hand and asked for food by saying in Spanish “Me dás?” (“Would you give me some?”). Both donors always performed the same behavior: when asked for food, they gave a corn flake to the beggar (see Figure 1A). The beggar always took the food but reacted differently to each donor. When interacting with the “positive” donor, the beggar ate the corn flake, and, while facing the donor, said in Spanish “Qué rico!” (“So tasty!”). When interacting with the “negative” donor, the beggar put the corn flake back onto the plate, said in Spanish “Qué feo!” (“So ugly!”), and turned his back to the donor (see Figure 1B). In group G, when the beggar approached a donor, he extended his hand without saying anything. The beggar always received a corn flake from the donors, and he either accepted or rejected the corn flake depending on the donor (positive versus negative). When he accepted the corn flake from the positive donor, he ate it while facing her. In contrast, when he rejected the corn flake from the negative donor, he put it back onto the plate and turned his back to her. Thus, the only difference between groups GV and G was that the beggar never spoke in the latter. In group V, the beggar approached each donor and said the same words that in group GV, though without hand or postural gestures. This meant that the beggar did not extend his hand and thus did not receive corn flakes in group V. Nevertheless, the beggar said “Me dás?” to both donors, and then said “Qué rico!” and “Qué feo!” to the positive and the negative donors, respectively (though he did not turn his back to the negative donor). Therefore, groups GV and V were equivalent in terms of vocalizations, though they differed in terms of the beggar’s hand gestures and body postures, and thus also on whether the beggar had access and ate corn flakes. In all groups, the positive and the negative donors were randomly chosen for each session (i.e., for each subject), and each donor was treated consistently as either positive or negative throughout a session.

After completing the six interactions with the donors (which took approximately 5 min), the beggar left the room, and then, the dog was released. The dog had 10 seconds to choose between the donors, who did not respond to the dog in any way (preliminary observations showed that after this time, non-rewarded dogs left the main scene and started exploring a larger area or simply lost interest). The donor whom the dog first approached (either the positive or the negative) was registered as dependent variable, which was defined as the dog being closer than 75 cm to one of the donors with the head oriented towards her. If the dog did not show a preference within 10 sec, we computed “no choice”. We also measured gaze duration (number of frames) towards each donor’s face using a frame by frame assessment (3 frames/s) of the video recordings. Gaze behavior was defined as the orientation of the dog’s head/nose toward the human face.

### Data Analysis

All video recordings of sessions were watched by two independent observers: the last author, MB, and an assistant. The assistant only watched the choice period of sessions but not the interactions between the beggar and the donors (i.e., the demonstrations), meaning that she was unaware of who the positive and the negative donors were on a particular session. Inter-observer reliability was 100% for the choice measure, and Spearman Rank Order correlations of observers’ records of gaze duration towards the positive and the negative donors showed R-values of 0.985 and 0.942 (both *Ps* < 0.001), respectively. For each group (GV, G, and V), we used a binomial test to compare the number of dogs who chose the positive (over the negative) donor against the number expected by chance (i.e., 50% of the sample). Pair-wise comparisons of the proportion of choices for the positive donor in groups GV, G, and V were done using calculated *Z-scores*. We used one-tailed tests to compare groups, because the direction of predictions was clear: we expected better discrimination of the beggar’s reactions in group GV than in groups G and V because of the combined cues in the former, and we expected better discrimination in group G than in group V because the beggar ate corn flakes (which presumably called the dogs’ attention) in the former but not in the latter group. Because gaze durations towards the positive and the negative donors were not normally distributed (*Shapiro-Wilk test*, both *W*s < 0.90, *Ps* < 0.001), we used two-tailed non-parametric tests to analyze these data. We used *Wilcoxon Matched Pairs tests* to compare gaze duration towards the positive donor versus the negative donor in each group, and *Krukal-Wallis tests* to make comparisons across groups. The alpha value was set at 0.05. We used the Holm-Bonferroni method to account for the effect of multiple comparisons between groups on the probability of a Type I error.

### Results

In groups GV, G, and V, eight, eleven, and eight subjects were discarded, respectively, because they did not make a choice. Therefore, data analyses were done with 15 in each group.

**Figure 2:**
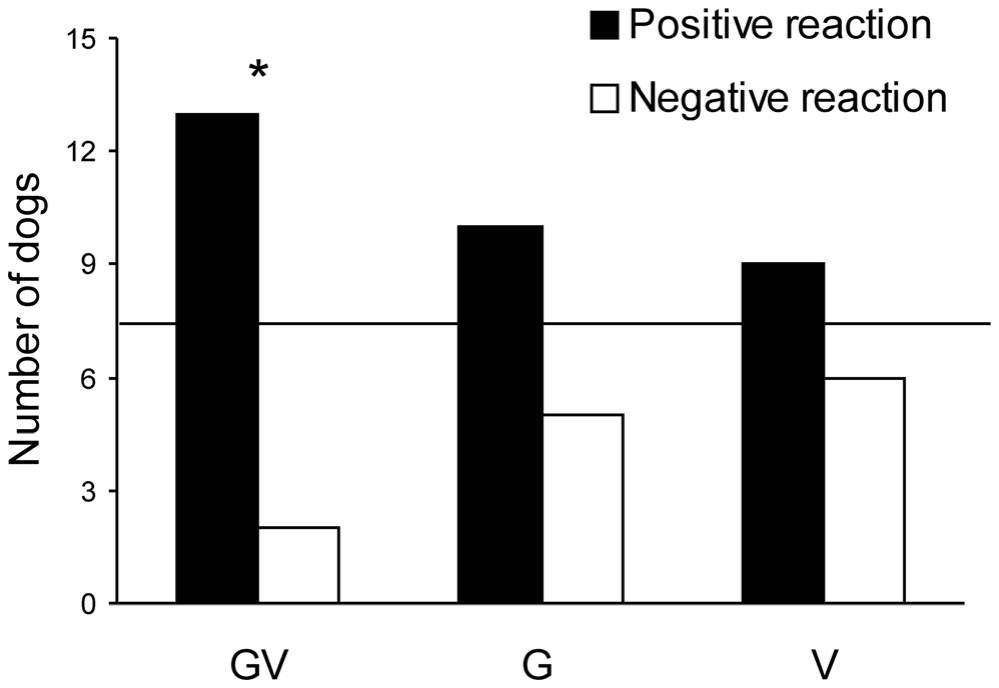
Frequency of choices for the positive and the negative donors as a function of group. Groups differed on whether the beggar’s reaction to the donors involved gestural and verbal cues (GV), gestural cues alone (G), or verbal cues alone (V). The horizontal line in the middle of the figure denotes the 0.50 chance level. * *P*<0.05, two-tailed binomial test.

Figure 2 shows the frequency of choices for the positive and the negative donors in groups GV, G, and V. In group GV, 13 out of 15 dogs chose the donor associated with the positive reaction of the beggar. A binomial test shows that this proportion is significantly different than expected by random choice (*P*<0.004). Only 10 out of 15 dogs, and 8 out of 15 dogs chose the positive donor in groups G and V, respectively (binomial tests: both *Ps* > 0.10). These last results indicate that dogs’ performance in groups G and V did not significantly depart from chance levels (see Figure 2). In addition, we found some evidence that the proportion of choices for the positive donor varied across groups. The difference between groups GV (87%) and V (53%) was marginally significant (corrected α = 0.017, *Z* = 1.99, *P* = 0.02), though the difference between groups G (67%) and V (53%) was non-significant (*Z* = 0.74, *P* = 0.23), and the difference between groups GV (87%) and G (67%) was not significant either (*Z* = 1.29, *P* = 0.09).

In terms of gaze duration (number of video frames), dogs in group GV looked towards the positive donor significantly longer than towards the negative donor (median ± 1 quartile; positive donor: 4.29 +2.97 –3.63; negative donor: 0.33 +2.31 –0.33; *Z* = 2.12, *P* = 0.03). In groups G and V, the comparison of gaze duration towards each donor did not reach statistical significance (group G: positive donor: 1.65 +4.62 –1.65; negative donor: 0 s +1.65 –0; *Z* = 1.60, *P* = 0.11; group V: positive: 1.32 + 1.65 – 1.32; negative donor: 2.64 +.99 –2.64; *Z* = 1.06, *P* = 0.29). Last, *Krukal-Wallis tests* showed that differences across groups were not significant for gaze duration towards the positive or the negative donor (positive donor, *K* = 4.10, *df* = 2, *P* = 0.13; negative donor, *K* = 3.50, *df* = 2, *P* = 0.17).

### Discussion

In this experiment, we showed that dogs in group GV were capable of developing a preference for people based on eavesdropping, that is, by watching interactions among them. This corroborates previous findings by Rooney and Bradshaw [22], Kundey et al. [23] and Marshall-Pescini et al. [24] who showed that dogs are capable of making indirect inferences of reputation. Here, however, we found, that dogs could develop a preference between demonstrators based, not on their behavior, but on the reaction that an interacting person (the beggar) showed towards them. In fact, dogs seemingly needed multiple cues to develop a preference for the positive donor. The preference was clear in group GV in which subjects counted on verbal and gestural cues and on the beggar eating during the positive demonstrations, but disappeared when fewer cues were available in groups G and V. The comparison with group V should, nonetheless, be taken with care because the beggar did not eat during demonstrations in this group, and that feature may have reduced dogs’ attention to the interactions relative to groups GV and G.

The indirect attribution of reputation to donors based on the beggar’s reaction in group GV implies a sequence of information processing stages. First, it implies the discrimination of the beggar’s positive and negative reactions. This assumption is consistent with studies showing that dogs are capable of discriminating some human emotional expressions [12]–[16]. Second, it may involve the association of the beggar’s reaction with the corresponding donor, even when both donors displayed the same behaviors. Last, it requires remembering the learned association at the moment of choice (when the beggar was not present anymore), which would lead them to prefer and approach the positive over the negative donor.

Alternatively, dogs could have associated the beggar’s reaction with the place where donors stood (a phenomenon of local enhancement), but not with the donors themselves. This alternative explanation still relies on dogs’ discrimination of the beggar’s positive and negative emotional reactions, but would not imply any attribution of reputation to the donors. Kundey and collaborators dealt with the problem of local enhancement by making their demonstrators switch places before the dog could choose between them (experiment 6, [23]). In experiment 1b, we followed a procedure similar to that of Kundey et al. [23] to control for local enhancement in a protocol with the features used in group GV of the present experiment 1a.

## Experiment 1b

In this experiment, dogs observed interactions between the beggar and the donors which were similar to those of group GV in experiment 1a, with the difference that donors switched places three times in between demonstrations. This procedure precluded an unambiguous association between the beggar’s reactions and a place. Hence, a preference for the positive donor would indicate that dogs associated the beggar’s positive reaction with that donor (and/or associated the negative reaction to the other donor).

### Methods

**Subjects.** We tested 23 adult domestic dogs of (mean ±1 SD) 4.51±3.13 years old. There were 10 females and 13 males from diverse breeds (2 Labrador Retrievers, 2 Golden Retrievers, 2 German Shepherds, 2 Argentine Dogos, 1 Boxer, 1 Setter, 1 Bloodhound, 1 Shiba Inu, 1 Beagle, 1 Bull Terrier, and 9 of mixed breeds). The other unspecified conditions were identical to those of experiment 1a.

**Procedure.** The experimental protocol was similar to that used for group GV in experiment 1a, with the following difference: in order to avoid the development of a preference for a place based on the beggar’s reactions, donors switched places after two approaches by the beggar (one to the positive and one to the negative donor). This meant that donors changed location three times in a session, and that the starting position for a particular donor was different from her last position. The donor at which the beggar started was counterbalanced across dogs. After the beggar’s sixth approach, he left the room, the donors did their last switch, and then the dog was released and could choose.

### Results

Five dogs were discarded because they did not make a choice. Therefore, data analyses were done with 18 subjects.

Nine dogs chose the donor associated with the beggar’s positive reaction, and the other nine dogs chose the donor associated with the beggar’s negative reaction. A binomial test shows that this proportion is not significantly different from chance (*P* = 0.18). Besides, gaze duration (number of video frames) towards the positive donor and the negative donor did not significantly differ (median ± 1 quartile; positive donor: 0.825+3.13 –0.49; negative donor: 2.475 ±2.145; *Z* = 0.15, *P* = 0.88).

### Discussion

Results from experiment 1b suggest that dogs could not associate the beggar’s positive and negative emotional reactions to the corresponding donors when donors switched places in between demonstrations. This negative result might be the consequence of confusion by the dogs (because the donors switched places many times) and insufficient experience with the situation. Indeed, dogs in the local enhancement control in the study by Kundey et al. experienced 10 demonstrations [23], whereas subjects in this experiment only observed six demonstrations (this was setup with the goal of making all groups equivalent in terms of the number of trials). Alternatively, the positive results of group GV in experiment 1a might have been the consequence of local enhancement. According to this possibility, subjects could have associated the beggar’s emotional reactions to the places where donors stood (left or right), but not to the donors themselves in experiment 1a. With the goal of evaluating this hypothesis we ran experiment 1c.

## Experiment 1c

In this experiment, donors were replaced by two high platforms on top of which the beggar placed the bowls with food. The beggar performed the same behaviors as in group GV of experiment 1a, though without interacting with the donors (indeed, there were no donors involved in the scene). If results from group GV relied on an association between the beggar’s reactions and the corresponding places (left and right), we would expect dogs in experiment 1c to choose the platform associated with the positive reaction.

### Methods

**Subjects.** We tested 27 adult domestic dogs of (mean ±1 SD) 2.93±1.67 years old. There were 11 females and 16 males of diverse breeds (3 Labrador Retrievers, 2 Jack Russell Terriers, 1 Golden Retriever, 1 Poodle, 1 Schnauzer, 1 Greater Swiss Mountain Dog, and 18 of mixed breeds). Unspecified conditions were identical to those of experiment 1a.

**Procedure.** In this experiment, there were no donors in the demonstrations. The beggar presented the plates with food to the dog, and then put each on top of a platform (100 cm high). Then, the beggar performed the same behaviors as in group GV, thus approaching the “positive” platform and the “negative” platform three times each. In the positive platform, the beggar asked for food, took a corn flake, ate it, and said “So nice!” while facing the bowl with food. In the negative platform, the beggar asked for food, took a corn flake, said “So ugly!”, returned it to the bowl, and turned his back to the negative platform. After the sixth approach, the beggar left the room, and then, the dog was released and could choose between the platforms.

### Results

Nine dogs were discarded because they did not make a choice. Therefore, data analyses were done with 18 subjects.

Only 8 dogs chose the positive platform, whereas the other 10 dogs chose the negative platform (binomial test, *P* = 0.17). In addition, 11 out of the 18 dogs chose the platform last visited by the beggar, but this was not significantly different from chance either (binomial test, *P* = 0.12). Therefore, we could not find any rule that dogs could have followed and that can thus explain their seemingly random preferences in experiment 1c.

### Discussion

Results from experiment 1c indicate that dogs were not able to make an association between the beggar’s reactions and the place where they occurred. These results have implications for the presumed mechanism underlying dogs’ choices in group GV in experiment 1a (i.e., the preference for the donor associated with the beggar’s positive reaction): first, it helps discard the possibility that dogs made an inference about the quality of the food in each side based on the beggar’s reactions; and second, it suggests that local enhancement could not have been the sole factor responsible for dogs’ preference, because the interaction between the beggar and the donors was a necessary element to obtain nonrandom choices as well.

## General Discussion

In the present study, we found that dogs could choose which donor to beg food from based, not on the behavior of the target individuals (the donors in our protocol), but on the reaction that an interacting person (the beggar, who was absent at the time of choice) showed towards them. This finding may indicate a level of subtlety in dogs’ eavesdropping not found before. The originality of present results relates, first, to dogs’ discrimination of the beggar’s positive and negative emotional reactions, and second, to the seeming association of such reactions with the interacting partners and/or with the places in which donors stood. These findings are consistent with previous studies in the literature showing that dogs rely on different aspects of people’s behavior to choose who to approach. For example, dogs preferred to approach a person who acted as if paying attention to them over a person who acted inattentive or distracted [27]–[30]. Dogs also preferred a person who signaled where the food was over a person who signaled an empty location in an object-choice task [31]. Overall, this study contributes to the description of dogs’ understanding of human communicative and social cues. Such “reading” of human signals could be crucial for dogs given that they typically depend on humans to have access to valuable resources such as food, water, and shelter.

In the context of present findings it is interesting to enquire about the cues dogs may have relied on to choose between donors. Altogether, the results of the experiments described in this manuscript allow us to conclude that:

- dogs discriminated between the beggar’s positive and negative emotional expressions, though subjects needed both gestural and verbal cues in order to reliably choose the positive donor (group GV in experiment 1a);
- the fact that dogs chose randomly when donors switched places in between demonstrations in experiment 1b suggests, first, that the place where donors stood could have played a role in experiment 1a, and, second, that dogs may find it difficult to spontaneously discriminate between unfamiliar people (at least, with the few trials used in the present protocol); and,
- an association of the beggar’s gestural and verbal reactions with a place or an object (a platform instead of a donor) was insufficient for subjects to develop a preference in experiment 1c, thus suggesting that the social interaction between the beggar and the donors was a necessary component of the observed scene for dogs to achieve a performance different from chance.

In sum, the successful discrimination between the donors found in group GV in experiment 1a suggests that dogs relied on multiple cues. Indeed, the absence of any of the above-mentioned cues impaired dogs’ discrimination in all the other experimental conditions implemented in this study.

In terms of the mechanism underlying dogs’ choices in present experiments, it is worth discussing different versions of the local enhancement hypothesis. One possibility is that local enhancement played a role through the association of the beggar’s eating behavior with either the positive donor and/or the place in which the positive donor stood. Several facts however speak against the possibility of a donor-food and/or a place-food association underlying dogs’ choices. To begin with, the frequency of choices for the positive donor was different from chance only in group GV in experiment 1a, but not in group G, while the eating behavior of the beggar was the same in both groups. Moreover, dogs in group GV gazed significantly longer towards the positive than the negative donor, but this did not happen in group G. In addition, one has to have in mind that both donors were eating at a regular pace as part of the protocol. This contrasts with the procedures in the studies by Kundey et al. [23] and Marshall-Pescini et al. [24] in which local enhancement was also a relevant concern. In their experiments food was given by only one of the demonstrators, leading to the possibility of a place-food association. This issue was minimized in the present study by the fact that both donors performed exactly the same food-related behaviors. Last, data from experiment 1c in which the beggar ate when he picked food from one platform but did not eat when he picked food from the other platform did not show any evidence of local enhancement (dogs did not develop any preference). Still, it is possible that the consistent pairing between the beggar’s eating behavior and a place may have served as one of several cues that dogs used.

Kundey and collaborators dealt with the problem of local enhancement by making demonstrators switch places before the dog’s choice (experiment 6, [23]). When we followed a similar control procedure in experiment 1b, dogs chose randomly between donors. The implications are that the main finding of group GV in experiment 1a may have depended on dogs forming an association of the beggar’s reactions, not with the donors, but with the places where the donors stood. This interpretation could be taken as an eavesdropping version of local enhancement because choices would have still relied on the discrimination of the beggar’s positive and negative reactions and the use of social information. However, when we tested this idea in experiment 1c (i.e., the beggar showed his emotional reactions towards platforms instead of towards people, thus removing the social component from the scene), we did not find any evidence of a systematic place preference. The presence of the donors (a social component attached to the beggar’s behavior) was apparently required for subjects to learn the discrimination between the beggar’s positive and negative reactions or the association of the reactions with other elements of the scene, such as people or places. It is possible that the social interactions called dogs’ attention to the scene, thus allowing them to learn the association between the beggar’s reactions and the corresponding donors and/or places. Indeed, this interpretation is consistent with results from the study by Marshall-Pescini et al. [24] who found an effect of demonstrators’ behavior on dogs’ preferences when the demonstrators interacted with a third party, but not when they performed the same behaviors alone.

Another concern with the results from group GV in experiment 1a is whether inadvertent cues by the donors might have guided subjects at the time of choice (equivalent to a “Clever Hans” effect). Though we cannot fully discard this alternative explanation, if it was true, dogs in all groups should have performed successfully on the task. However, only dogs in the group with both gestural and verbal cues in experiment 1a did perform above chance levels, whereas dogs with gestural or verbal cues alone did not, and neither did the group with gestural and verbal cues in which the donors switched places in between demonstrations. This suggests that dogs relied on compound cues to distinguish and be guided by the beggar’s emotional reactions. The fact that dogs in the group with verbal cues alone performed randomly in the measures taken in the choice period (choice and gaze duration) is noteworthy because it differs from the results obtained by Marshall-Pescini and collaborators [24] whose dogs were able to use verbal cues from the demonstrations to guide their choices (though their dogs also performed better when they counted on both gestural and verbal cues than when they counted on verbal cues alone). This difference between studies may stem from the fact that in the present experiment 1a the beggar never ate in demonstrations of the verbal group, whereas in the study by Marshall-Pescini et al., eating by the beggar did occur in interactions with the positive donor. This comparison suggests that the mere verbal interaction between donor and beggar, despite differing in emotional tone, may not have any effect on dogs’ behavior if there is no exchange of food involved.

An outcome of our study worth discussing is the fact that a large number of dogs had to be discarded because they did not make a choice. We believe that this could be related to subjects’ individual differences on additional factors such as the degree of sociability and the level of motivation for food. Indeed, we observed signs of fear (probably because the experimental assistants were unknown to them) or simply inattention to the experimental interactions in most of the dogs that were discarded. Marshall-Pescini and collaborators mentioned similar problems in their study [24], and, in fact, this interpretation is consistent with previous findings from our research group that showed that dogs with low sociability are less persistent in their communicative responses towards people [32]. In this sense, dogs’ degree of sociability and level of motivation for food may be relevant factors to measure and take into account in future studies.

In conclusion, we showed that dogs have the capacity to recognize subtle human expressions which may signal others’ disposition to share valuable resources. To do so, they seemingly use information from multiple cues. In other studies in the same line of research, such as Kundey et al.’s [23] and Marshall-Pescini et al.’s [24], authors claim that dogs have the ability to attribute reputation, namely to assign value to people based on observing their past interactions. Even when dogs were successful in making the discrimination between donors in this study, present data suggest that they did not rely on the donors’ physical features to do that. Subjects may have just associated the beggar’s reactions to the locations where donors stood. Arguably, dogs might prioritize spatial cues over the physical characteristics of the persons involved when processing the information from a scene. Future studies could assess whether more or alternative experiences with the donors (e.g., pre-exposure) could help subjects achieving a more proficient discrimination of people’s physical appearance, and the impact of this on dogs’ reputation tracking performance.

Finally, the present study does not allow us to make a conclusive inference about how subjects processed and integrated the multiple cues they relied on and about the origin of these socio-cognitive abilities. Future research should focus on unraveling the relative contribution of innate and learning processes on the development of eavesdropping in dogs. The comparison of dogs with different levels of human contact, such as, for example, shelter versus family dogs, could be used with this goal in mind.
